# Microsporidian Genomes Harbor a Diverse Array of Transposable Elements that Demonstrate an Ancestry of Horizontal Exchange with Metazoans

**DOI:** 10.1093/gbe/evu178

**Published:** 2014-08-28

**Authors:** Nicolas Parisot, Adrian Pelin, Cyrielle Gasc, Valérie Polonais, Abdel Belkorchia, Johan Panek, Hicham El Alaoui, David G. Biron, Émilie Brasset, Chantal Vaury, Pierre Peyret, Nicolas Corradi, Éric Peyretaillade, Emmanuelle Lerat

**Affiliations:** ^1^Clermont Université, Université d’Auvergne, EA 4678 CIDAM, Clermont-Ferrand, France; ^2^CNRS, UMR 6023, LMGE, Aubière, France; ^3^Canadian Institute for Advanced Research, Department of Biology, University of Ottawa, Ontario, Canada; ^4^Clermont Université, Université d’Auvergne, Laboratoire “Microorganismes: Génome et Environnement,” Clermont-Ferrand, France; ^5^Clermont Université, Université d’Auvergne, Clermont-Ferrand, France, Inserm; U 1103, Clermont-Ferrand, France, CNRS; UMR 6293, Clermont-Ferrand, France; ^6^Université de Lyon; Université Lyon 1; CNRS, UMR 5558, Laboratoire de Biométrie et Biologie Évolutive, F-69622 Villeurbanne, France

**Keywords:** microsporidia, transposable elements, diversity, genome evolution, horizontal transfers

## Abstract

Microsporidian genomes are the leading models to understand the streamlining in response to a pathogenic lifestyle; they are gene-poor and often possess small genomes. In this study, we show a feature of microsporidian genomes that contrasts this pattern of genome reduction. Specifically, genome investigations targeted at *Anncaliia algerae,* a human pathogen with a genome size of 23 Mb, revealed the presence of a hitherto undetected diversity in transposable elements (TEs). A total of 240 TE families per genome were identified, exceeding that found in many free-living fungi, and searches of microsporidian species revealed that these mobile elements represent a significant portion of their coding repertoire. Their phylogenetic analysis revealed that many cases of ancestry involve recent and bidirectional horizontal transfers with metazoans. The abundance and horizontal transfer origin of microsporidian TEs highlight a novel dimension of genome evolution in these intracellular pathogens, demonstrating that factors beyond reduction are at play in their diversification.

## Introduction

Microsporidia are a group of obligate intracellular parasites composed of over 1,500 species and over 187 genera (Vavra and Lukes 2013), which have been recently associated with a phylum closely related to the fungal kingdom, the Cryptomycota (James et al. 2013). These organisms are ubiquitous and able to infect potentially all animal phyla, particularly insects but also humans, so these parasites are considered both medically and ecologically relevant (Vavra and Lukes 2013). Obligate intracellular lifestyle in microsporidia resulted in severe reduction of their genomes and cells complexity, which, for example, now lacks conventional mitochondria and Golgi apparatus (Didier et al. 2009; Vavra and Lukes 2013). Microsporidian genomes sequenced to date encode few known protein coding genes compared with other eukaryotes, so most members have a minimal metabolic networks and rely on several key transporters to stealing metabolites from their hosts’ cell, including molecular sources of energy (Nakjang et al. 2013). Interestingly, although the biochemical repertoire of microsporidia is universally reduced, the genome size within the group can vary over 10-fold (2.3–24 Mb) (Corradi et al. 2009, 2010), so variation in genome size in this group is not necessarily correlated with significant improvements in metabolic capabilities, but rather with an expansion in the size of noncoding regions (i.e., larger intergenic regions), as well as the number of transposable elements (TEs) and other DNA repeats (Corradi et al. 2009; Peyretaillade et al. 2012; Pan et al. 2013). Indeed, TEs are found and characterized in microsporidia with comparatively large genomes, including *Nosema* spp, *Hamiltosporidium tvaerminnensis*, and *Anncaliia algerae*, but are typically absent in species with smaller genomes, such as *Enterocytozoon bieneusi* and species in the genus *Encephalitozoon* (Katinka et al. 2001; Akiyoshi et al. 2009; Corradi et al. 2010; Keeling et al. 2010; Pombert et al. 2012). To date, no studies have looked at the overall diversity of TEs in these pathogens, and their potential impact on their ecology and evolution.

TEs are repeated sequences that usually represent a substantial part of the fungal (3–20%) and metazoan (0.13–50%) genomes (Daboussi and Capy 2003; Hua-Van et al. 2005; Wicker et al. 2007; Wang et al. 2010; Sun et al. 2012). Among these, two major classes are generally recognized, including Class I elements (i.e., retrotransposons), which can move through a reverse-transcribed RNA intermediate and are subdivided into two subclasses according to the presence or absence of Long Terminal Repeat (LTR) sequences at their extremities, and Class II elements (i.e., DNA transposons) that can transpose directly under the form of DNA intermediate (Wicker et al. 2007). In most genomes, TEs can rapidly multiply, resulting in several structural changes, including chromosomal rearrangements, pseudogenizations, and gene shuffling (Biemont 2010) that are often deleterious. In few cases, however, the presence of TEs can also benefit an organism, notably by modulating the expression of neighboring genes or by creating genomic diversity in regions that are important in host–parasite interactions—that is, antigens, effectors (Raffaele and Kamoun 2012).

TEs also have a high propensity for horizontal transfers (HTs) (Loreto et al. 2008); an HT is the nonsexual exchange of DNA between organisms that are not necessarily related. Similar events involving TEs were reported from a variety of lineages, including plants, mammals, insects, and unicellular eukaryotes such as microsporidia (Yoshiyama et al. 2001; Steglich and Schaeffer 2006; Laha et al. 2007; Fortune et al. 2008; Hecht et al. 2010; Heinz et al. 2012; Wallau et al. 2012; Ivancevic et al. 2013; El Baidouri et al. 2014; Zhang et al. 2014), but little is known about the cellular mechanisms involved in such transfers (Gilbert et al. 2010b). Nevertheless, some studies proposed that the intimacy of parasitism (direct host–parasite interaction) could promote HT of TEs across phyla like between vertebrates and invertebrates (Gilbert et al. 2010a; Gilbert et al. 2010b). In microsporidia, the genetic/cellular intimacy arising from intracellular parasitism has fuelled a number of HTs (Corradi and Selman 2013), but intriguingly only few of these have involved TEs (Heinz et al. 2012; Pan et al. 2013). The rarity of HTs involving TEs in these species may represent a real biological barrier to transfer, but could also reflect the poor annotation of these elements in genome sequences from this group because in the multicellular eukaryotes, the HT of TEs usually outnumbers those involving protein encoding genes (Schaack et al. 2010). To differentiate these two scenarios, and thus better understand the diversity and role of TEs in these parasites, we conducted an exhaustive search for TEs across all publicly available microsporidian genomes, with a particular focus on a species with one of the largest genomes known in the group (23 Mb), the human pathogen *An. algerae* (Belkorchia et al. 2008). Our analyses demonstrated that TE families can be present in surprisingly large numbers in microsporidia, and that some of these have been involved in HT between the animals (possibly the hosts) and microsporidia; possibly in both directions.

## Materials and Methods

### Sequence Data from Complete Genomes

#### Genome Data from *An. algerae*

The contig sequences obtained from *An. algerae* (Peyretaillade et al. 2012) were searched using BLASTX (Altschul et al. 1997) against the nonredundant GenBank database to determine matches to proteins from TEs. We selected 1,785 contigs containing domains supposedly corresponding to TE sequences and clustered them using BLASTClust (ftp://ftp.ncbi.nih.gov/blast/documents/blastclust.html, last accessed September 1, 2014) with the following parameters: 90% of identity and 60% of coverage. We obtained 903 clusters (size ranges from 1 to 24 sequences). For each cluster of size superior to 1, the sequences were aligned using MUSCLE (Edgar 2004) and used to build a consensus. This consensus was then used as query for a BLASTN against all the *An. algerae* contigs to retrieve sequences with over 90% of identity over a length of more than 200 bp. The contig sequences were then aligned using MUSCLE and a new consensus sequence was reconstructed to likely represent a complete TE sequence. For each consensus, the presence of Open Reading Frame(s) (ORF) and the presence of LTR or Terminal Inverted Repeat (TIR) sequences were detected using ORF Finder (Sayers et al. 2011) and bl2seq (Altschul et al. 1997), respectively. In total, 240 different consensus corresponding to different TE families from various types (LTR retrotransposons and DNA transposons) were obtained (supplementary table S1 and file S1, Supplementary Material online). The occurrences of each family were determined using RepeatMasker (Smit et al. 1996–2010) on the sequenced genomes of *An. algerae* with the consensus sequences used as library, and the copy numbers were computed using the tool One_code_to_find_them_all (Bailly-Bechet et al. 2014) (supplementary table S2, Supplementary Material online).

#### Data from Other Microsporidia

Available microsporidian genomes (16) were retrieved: *Edhazardia aedis* USNM 41457 (AFBI00000000.2), *Encephalitozoon cuniculi* EC1 (AEWD00000000.1), *Enc. cuniculi* EC2 (AEWQ00000000.1), *Enc. cuniculi* EC3 (AEWR00000000.1), *Enc. cuniculi* GB-M1 (AL391737.2; AL590442.1–AL590451.1), *Encephalitozoon hellem* ATCC 50504 (CP002713.1–CP002724.1), *Encephalitozoon intestinalis* ATCC 50506 (CP001942.1–CP001952.1), *Encephalitozoon romaleae* SJ-2008 (CP003518.1–CP003530.1), *Ent. bieneusi* H348 (NZ_ABGB00000000.1), *Ham. tvaerminnensis* OER-3-3 (ACSZ00000000.1), *Nematocida parisii* ERTm1 (AEFF00000000.2), *Ne. parisii* ERTm3 (AEOO00000000.1), *Nematocida* sp. 1 ERTm2 (AERB00000000.1), *Nosema antheraeae* YY (http://silkpathdb.swu.edu.cn/silkpathdb/ftpserver, last accessed September 1, 2014), *Nosema apis* BRL 01 (ANPH00000000.1), *Nosema bombycis* CQ1 (ACJZ00000000.1), *Nosema ceranae* BRL01 (NZ_ACOL00000000.1), *Spraguea lophii* 42_110 (ATCN00000000.1), *Trachipleistophora hominis* (ANCC00000000.1), and *Vavraia culicis* subsp. floridensis (AEUG00000000.1). Using the reconstructed consensus TE sequences from *An. algerae*, a TBLASTX (Altschul et al. 1997) was performed (*E* value threshold of 1e^−^^5^ and low-complexity filter disabled) against microsporidian genomes. For each species, TE consensus were then reconstruct as described for *An. algerae* (see above, see table 1). To identify highly variable or additional subclass of TEs, an exhaustive search of TEs has been carried out with TransposonPSI software (http://transposonpsi.sourceforge.net/, last accessed September 1, 2014).

**Table 1:** Number of Families for Each TE Class in Microsporidian Species

Microsporidian Species	Genome Size (Mb)	Known Hosts	Non-LTR Retrotransposons (LINE)	LTR Retrotransposons	DNA Transposons				Total
					*Helitron*	*Mariner/Tc1*	*Merlin*	*piggyBac*	
*Anncaliia algerae* (Peyretaillade et al. 2012)	23	Mammals, insects	4	97	/	16	120	3	240
*Edhazardia aedis*^a^ (Williams et al. 2008)	not defined	Mosquitoes	4	28	/	/	1	/	33
*Encephalitozoon cuniculi* (Katinka et al. 2001)	2.9	Mammals	/	/	/	/	/	/	/
*Encephalitozoon intestinalis* (Corradi et al. 2010)	2.3	Mammals	/	/	/	/	/	/	/
*Encephalitozoon hellem* (Pombert et al. 2012)	2.5	Mammals, birds	/	/	/	/	/	/	/
*Encephalitozoon romaleae* (Pombert et al. 2012)	2.5	Insects	/	/	/	/	/	/	/
*Enterocytozoon bieneusi* (Akiyoshi et al. 2009; Keeling et al. 2010)	<6	Humans	/	/	/	/	/	/	/
*Hamiltosporidium tvaerminnensis* (Corradi et al. 2009)	≤24.2	Daphnia	12	5	4	1	/	3	25
*Nematocida parisii* (Cuomo et al. 2012)	<4.1	Nematodes	/	4	/	1	/	1	6
*Nematocida* sp. (Cuomo et al. 2012)	4.7	Nematodes	1	6	/	1	/	/	8
*Nosema antheraeae* (Pan et al. 2013)	9.3–9.5	Insects	6	7	/	6	4	1	24
*Nosema apis* (Chen et al. 2013)	8.5	Insects	15	5	/	/	/	5^b^	25
*Nosema bombycis* (Pan et al. 2013)	15–16	Insects	11	14 ^c^	1	15^d^	9	7	57
*Nosema ceranae* (Cornman et al. 2009)	<7.86	Insects	4	3	3	1	6	5	22
*Spraguea lophii* (Campbell et al. 2013)	6.2–7.3	Fishes	1	3	/	2	6	/	12
*Trachipleistophora hominis* (Heinz et al. 2012)	11.6	Humans, mosquitoes	9	1	/	/	/	1	11
*Vavraia culicis*^a^	6.12	Mosquitoes	/	1	1	/	/	1	3

^a^Data from the Microsporidia Comparative Sequencing Project, Broad Institute of Harvard and MIT (http://www.broadinstitute.org/, last accessed September 1, 2014).

^b^That were all already described in GenBank.

^c^Including eight that were already described in GenBank.

^d^Including two that were already described in GenBank.

### Reference Sequences of TEs

Phylogenetic analyses were performed using different eukaryotic protein sequences from reference TE elements obtained from the Repbase database (Jurka 2000) (supplementary table S3, Supplementary Material online). We performed BLASTN searches of each *An. algerae* consensus on the GenBank databases to add other TE sequences for the different tree reconstructions (supplementary table S4, Supplementary Material online). We used *piggyBac* previously described sequences (Sarkar et al. 2003; Pan et al. 2013) (supplementary table S5, Supplementary Material online). We used the ISFinder database (Siguier et al. 2006) to retrieve bacterial sequence for the *Merlin* phylogenetic analysis.

#### Domain Detection

The protein domains of each consensus were determined using the Pfam database version 27.0 (March 2013, 14,831 families; http://pfam.sanger.ac.uk/, last accessed September 1, 2014) (Punta et al. 2012) and the batch web CD-search tool (http://www.ncbi.nlm.nih.gov/Structure/bwrpsb/bwrpsb.cgi, last accessed September 1, 2014) (Marchler-Bauer et al. 2011).

#### In Silico Confirmation

TEs representing novel phylogenetic incongruences were validated in silico as being present in a contig belonging to the host genome rather than a contaminant. Most TEs are present in multiple copies in the genome, in such cases contigs were selected based on the proximity of microsporidian coding sequences to TEs. Paired-end reads from *Schmidtea mediterranea* and *Dendroctonus ponderosae* were mapped using Geneious (Geneious version R7 available from http://www.geneious.com/, last accessed September 1, 2014) against contigs harboring TEs (Dpon, *Mariner*12, *Merlin*7, 10, 2, and 4) and the nucleotide coverage was plotted to validate the presence of TE in the respective host genome (supplementary fig. S5*A–F*, Supplementary Material online). For the TEs of *No. apis* and *Ap. florea,* reads consisting of mate pairs were mapped against contigs to validate the assembly of contigs (supplementary fig. S5*H*, Supplementary Material online).

### Tree Reconstruction and Sequence Analysis

For each superfamily, the protein sequences were aligned using MAFFT version 6 (Katoh et al. 2002). Uninformative columns in each of the alignments were removed using the trimAl algorithm (Capella-Gutierrez et al. 2009). In order to determine the amino acid evolution model to be used in our phylogenetic analysis, we analyzed each alignment with ProtTest (Darriba et al. 2011). This revealed the models LG+Г (LTR retrotransposons), LG+I+Г (*Mariner/Tc1*), Blosum62+I+Г (*Merlin*), and VT+Г (*piggyBac*) to best explain our data. Maximum-likelihood analysis was performed using PHYML v3.0 (Guindon et al. 2010) with 100 bootstrap replicates, whereas Bayesian analysis was performed using MrBayes v3.2.1 (Huelsenbeck and Ronquist 2001) for 5,000,000 generations and a burnin of 25%. In order to use the LG model for Bayesian analysis, we obtained the latest development version r851 of MrBayes (http://sourceforge.net/p/mrbayes/, last accessed September 1, 2014).

For the LTR-retrotransposon phylogenetic analyses, 50 complete consensus sequences and 10 incomplete consensus sequences that corresponded to nearly complete ORFs of polyproteins were used. Other microsporidian polyproteins consensus sequences were also included (12 consensus sequences from *No. bombycis*, 13 from *Ed. aedis*, 4 from *Ne. parisii*, 7 from *No. antheraeae*, 3 from *Nematocida* sp., 1 from *No. ceranae*, and 3 from *Sp. lophii*) as well as LTR-retrotransposon polyproteins and Pol proteins representative of the *Ty3/gypsy* and*Ty1/copia* groups (supplementary tables S3 and S4, Supplementary Material online)

For the *Merlin* phylogenetic analysis, 82 potentially complete consensus protein sequences over the 84 identified in *An. algerae* were selected; those having no complete ORF were removed. Transposase consensus sequences (34 over 36, two sequences were removed as too short), all microsporidian consensus sequences (six consensus sequences from *No. ceranae*, four from *Sp. lophii*, nine from *No. bombycis*, four from *No. antheraeae,* one from *Ed. aedis*) as well as several transposase proteins from reference *Merlin* elements from various organisms were also included (supplementary table S3, Supplementary Material online).

To build the *Mariner/Tc1* phylogenetic tree, 15 protein consensus sequences from *An. algerae Mariner/Tc1* elements and the one described as member of the *Tc5* family were selected as well as consensus sequences reconstructed for the other microsporidian species (15 consensus sequences from *No. bombycis*, 1 from *Sp. lophii*, 5 from *No. antheraeae*, and 1 from *No. ceranae*) and reference elements from various organisms (supplementary tables S3, S4, and S6, Supplementary Material online).

To reconstruct the evolutionary history of the *piggyBac* superfamilies, the three consensus sequences identified in *An. algerae*, the proteins from the consensus sequences identified in other microsporidian species (seven consensus sequences from *No. bombycis*, one from *T. hominis*, one from *No. antheraeae*, and three from *No. apis*) and 121 protein sequences from various organisms (supplementary tables S3, S4, and S6, Supplementary Material online) corresponding to reference elements and also to *piggyBac*-derived genes were used.

Phylogenetic inconsistencies of TEs clustered with homologs from very distantly related species were confirmed computing Ks values. We retrieved 26–43 orthologous genes between Microsporidia and involved metazoans to compute the mean Ks values using seqinR (Charif et al. 2005). Putative HT sequences sharing over 90% nucleotidic identity with metazoans or involving poorly annotated genomes were not considered for Ks calculations.

## Results

### TEs Can Be Very Diverse in Microsporidian Genomes

**Fig. 1.—:**
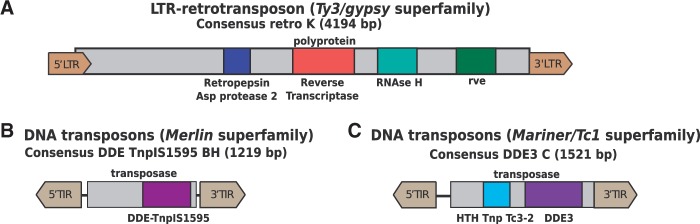
Structure of representative complete consensus sequences from the *Ty3/gypsy*, *Merlin*, and *Mariner/Tc1*superfamilies detected in the genome of *Anncaliia algerae.* (*A*) Structure of a *Ty3/gypsy* LTR retrotransposon; (*B*) structure of a *Merlin* DNA transposon; (*C*) structure of a *Mariner/Tc1* DNA transposon.

Analyses of the *An. algerae* genome, identified a diverse collection of TEs, and consensus sequences could be reconstructed for a total of 97 LTR retrotransposons, four non-LTR retrotransposons, and 139 DNA transposons (from the superfamilies’ *Mariner*/*Tc1*, *piggyBac*, and *Merlin*) (table 1; supplementary table S1 and file S1, Supplementary Material online). Representative structures of each superfamily for which complete consensus sequences were reconstructed are shown on figure 1. Because of the highly fragmented state of the *An. algerae* genome, only 50 of the 97 different LTR-retrotransposon consensus sequences could be fully reconstructed (i.e., with LTRs at both extremities and a potentially complete coding sequence; fig. 1*A*), whereas others were identified based on the presence of partial sequences or complete integrase domain (*rve*). For non-LTR retrotransposons, a total of four distinct families could be found, all of which encode for a specific reverse transcriptase CDD domain cd01650. In the case of the *Merlin* superfamily (DNA transposon), 120 consensus sequences could be reconstructed, 84 of which consisted in complete sequences with characteristic TIRs and a transposase coding sequence (880 bp on average). All of these 84 transposases displayed the DDE_Tnp_IS1595 domain typical of ISXO2-like transposases (fig. 1*B*). For The *Mariner/Tc1* superfamily of DNA transposons, 16 different consensus sequences could be reconstructed, 12 of them were complete based on the presence of identifiable TIRs at both extremities and a complete transposase coding gene (fig. 1*C*). With one exception, transposases displayed the two protein domains: A “helix-turn-helix” domain that specifies DNA binding and DNA recognition domain of the *Tc3* transposase (HTH_tnp_Tc3-2), and a DDE-3 domain characteristic from DDE transposase displaying the carboxylate residues responsible for the endonuclease activity of the protein (fig. 1*C*). The one exception included a transposase with a “helix-turn-helix” domain characteristic of *Tc5* family (HTH_Tnp_Tc5) linked to a DDE-1 endonuclease activity domain. Finally, we also reconstructed three consensus sequences corresponding to *piggyBac* elements, all of which encoded potentially complete transposase genes which displayed the domain DDE_Tnp_1_7 typical of *piggyBac* transposases, but for which no TIRs could be detected. The copy number of each family was also investigated along the *An. algerae* genome revealing that LTR retrotransposons and *Merlin* DNA transposons represent the most abundant families in this species (supplementary table S2, Supplementary Material online). Importantly, the results obtained using the same methodology based on publicly available data from two other *An. algerae* strains (NCBI [National Center for Biotechnology Information] BioProjects PRJNA188095 and PRJNA188094) resulted in very similar findings.

Our searches for TEs were also expanded to all 16 available genome sequences using two independent approaches (table 1). Specifically, all different consensus sequences retrieved from *An. algerae* were used as queries for BLAST-based searches against the 16 microsporidian genomes (see Materials and Methods), and this approach was complemented by a TransposonPSI analysis of these genomes (http://transposonpsi.sourceforge.net/, last accessed September 1, 2014) (table 1). These inspections revealed the presence of previously unrecognized TEs, with ORFs encoding for a helitron helicase-like domain (Pfam domain: 14214) in *No. bombycis*, *No. ceranae*, *Ham. tvaerminnensis*, and *V. culicis*. In total, our exploration of other available microsporidian genomes resulted in the identification of 33, 25, 24, 25, 57 and 22 TE families in the *Ed. aedis*, *Ham. tvaerminnensis*, *No. antheraeae*, *No. apis*, *No. bombycis* and *No. ceranae* genomes, respectively, whereas less than 15 families in total were detected in the other species (table 1).

### Phylogenetic Reconstructions Reveal Microsporidia-Specific Clades of TEs

**Fig. 2.—:**
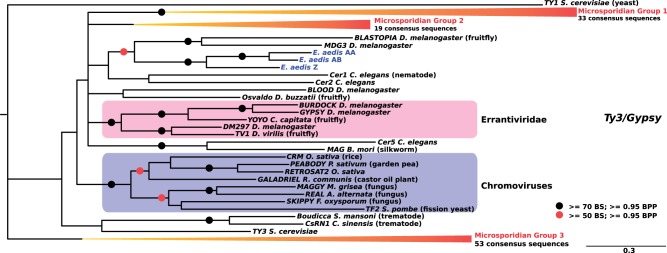
Phylogenetic tree of the LTR-retrotransposon class. Tree topology obtained using Bayesian analysis on the Pol proteins and rooted by the *Ty1* reference element from yeast. Black circles highlight nodes with bootstrap support (BS) higher than 70% and bayesian posterior probability (BPP) values higher than 0.95, whereas red circles indicate BS higher than 50% and BPP values higher than 0.95. Absence of red and black circles indicates BPP values above 0.50. The three groups of microsporidian sequences are indicated by the red collapsed clades. For the uncollapsed version of the tree, see supplementary figure S1, Supplementary Material online. Three consensus sequences from the microsporidia *Edhazardia aedis* are indicated in blue. Colored frames represent the well-identified genera corresponding to Errantiviridae and Chromoviruses.

**Fig. 3.—:**
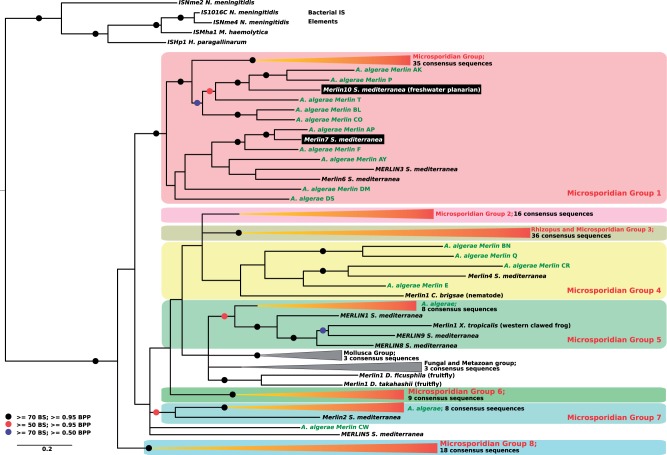
Phylogenetic tree of the *Merlin* superfamily. Tree topology was obtained using Bayesian analysis on transposase proteins and rooted by bacterial IS elements. Black circles highlight nodes with bootstrap support (BS) higher than 70% and bayesian posterior probabiliy (BPP) values higher than 0.95, red circles indicate BS higher than 50% and BPP values higher than 0.95, and blue circles indicate BS higher than 70% and BPP values higher than 0.50. Absence of red, black, and blue circles indicates BPP values above 0.50. The eight Microsporidian groups are indicated by coloured frames and collapsed red clades. For the uncollapsed versions of the tree, see supplementary figure S2, Supplementary Material online. Several consensus sequences from the microsporidia *An. algerae* are indicated in green. Putative HT sequences are shown in black rectangles.

**Fig. 4.—:**
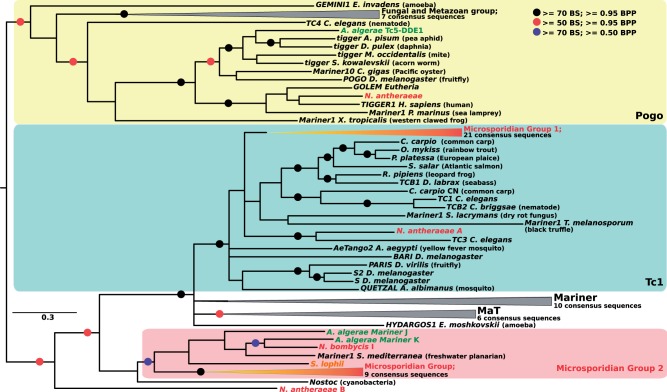
Phylogenetic tree of the *Mariner/Tc1* and *pogo* superfamilies. Tree topology was obtained using Bayesian analysis on transposase proteins and rooted by elements from the *pogo* superfamily. Black circles highlight nodes with bootstrap support (BS) higher than 70% and bayesian posterior probability (BPP) values higher than 0.95, red circles indicate BS higher than 50% and BPP values higher than 0.95, and blue circles indicate BS higher than 70% and BPP values higher than 0.50. Absence of red, black, and blue circles indicates BPP values above 0.50. The four classic groups from the Mariner/Tc1 superfamily (Mariner, Tc1, MaT, and pogo) are represented by colored frames and gray collapsed clades. The two Microsporidian groups are represented by red collapsed clades. Some sequences of the microsporidia are indicated in different text colors: green for *An. algerae*, red for *No. bombycis* and *No. antheraeae*, and orange for *S. lophii*. For the uncollapsed version of the tree, see supplementary figure S3, Supplementary Material online.

The evolutionary histories of the four largest microsporidian TE superfamilies were investigated using Maximum Likelihood and Bayesian analyses of their complete consensus sequences. These confirmed that the vast majority of microsporidian LTR retrotransposons correspond to elements from the *Ty3/gypsy* group (fig. 2 and supplementary fig. S1, Supplementary Material online), which separate into three main groups that seem to have specifically diversified in microsporidia (i.e., they do not contain elements from other eukaryotes; see, e.g., group 1 in supplementary fig. S1, Supplementary Material online). *Anncaliia algerae* LTR retrotransposons displayed a pattern of extreme diversification within three main groups (39 sequences in group 3, 13 and 8 sequences in group 1 and 2, respectively). A similar pattern of diversification is also found in *Ed. aedis,* although the total number of sequences is much smaller than in *An. algerae* (28 different families; table 1). For the *Merlin* superfamily, microsporidian sequences clustered into eight phylogenetic groups, three of which contained 72% of all consensus sequences (fig. 3 and supplementary fig. S2, Supplementary Material online), suggesting that diversity in *Merlin*-like sequences in *An. algerae* results from several independent events of diversification within each group. The four classic groups from the *Mariner/Tc1* superfamily (*Mariner, Tc1, MaT*, and *pogo*) are well supported in the phylogenetic tree (fig. 4 and supplementary fig. S3, Supplementary Material online). Nine consensus sequences of *An. algerae* appeared to be part of the *Tc1* group along with other sequences from other microsporidia, whereas the remaining six, along with other microsporidian sequences, formed an independent group at the basis of *Mariner*, *Tc1*, and *MaT* groups but also independent of the *pogo* group. The *An. algerae Tc5* consensus sequence (*An. algerae Tc5* DDE1) and the one identified in *No. antheraeae* clustered inside the *pogo* group, though not together (fig. 4 supplementary fig. S3, Supplementary Material online).

### Phylogenetic Incongruences for TEs Identified in Microsporidian and Metazoan Genomes

**Fig. 5.—:**
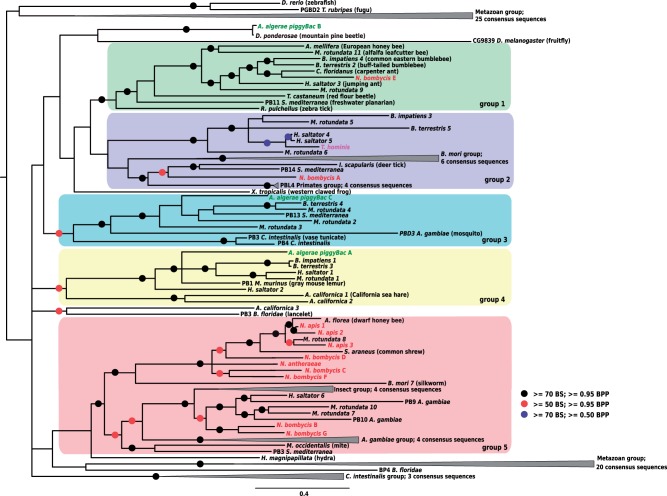
Phylogenetic tree of the *piggyBac* superfamily. Tree topology was obtained using Bayesian analysis on transposase proteins and rooted at midpoint. Black circles highlight nodes with bootstrap support (BS) higher than 70% and bayesian posterior probability values higher than 0.95, red circles indicate BS higher than 50% and BPP values higher than 0.95, and blue circles indicate BS higher than 70% and BPP values higher than 0.50. Absence of red, black, and blue circles indicates BPP values above 0.50. Colored frames represent five well supported clades. Some sequences have been groups inside collapsed gray clades. For the uncollapsed version of the tree, see supplementary figure S4, Supplementary Material online. The consensus sequences of microsporidia appear in text color: green for *An. algerae*, red for *No. bombycis*, *No. apis*, and *No. antheraeae*, and pink for *T. hominis*.

Our phylogenetic reconstruction revealed 17 instances where microsporidian TEs clustered with homologs from very distantly related species, notably arthropods and platyhelminthes, or vice versa. Nine of these are strongly supported (Bootstraps values [BS] and Bayesian Posterior Probability [BPP] support over 95%) by two or more nodes, and four are also backed by an extensive sequence identity. Compelling cases of phylogenetic incongruence include two *Merlin* DNA transposons from the planarian *Sc. mediterranea* (*Merlin7* and *Merlin10* in fig. 3) that cluster within clades otherwise exclusively composed of elements from *An. algerae*, and others involving *piggyBac* clustered with homologs from insects (e.g., *An. algerae*-*piggyBac*-A and *An. algerae*-*piggyBac*-B; fig. 5 and supplementary fig. S4, Supplementary Material online). Among these, the sequence *An. algerae*-*piggyBac*-B is particularly intriguing, as it shares over 98.86% of nucleotide identity with a sequence from the Pine beetle *D. ponderosae;* suggesting their very recent divergence. Other phylogenetic inconsistencies include some previously found by others, (i.e., a *piggyBac* element from *T. hominis* related to an ant *Harpegnathos saltator*, fig. 5 [Heinz et al. 2012] as well as three *No. apis* sequences [*N. apis* 1,2,3; fig. 5 and supplementary fig. S4, Supplementary Material online]). These sequences cluster and share an identity higher than expected with homologs from different bee species (respectively 94.18% and 99.20% nucleotide identity with the *Ap. florea*, and 90.32% identity with the *Megachile rotundata*; fig. 5). The number of synonymous substitutions per synonymous site (Ks) was computed for TEs with phylogenetic inconsistencies but sharing intermediate sequence identity with metazoan homologs. Additional evidence of HTs were found for *piggybac* elements from *No. bombycis* (mean Ks [*Camponotus floridanus* genes vs. *No. bombycis* genes] = 9.03 ± 2.52 vs. Ks [*N. bombycis E* vs. *C. floridanus*] = 1.92; mean Ks [*M. rotundata* genes vs. *No. bombycis* genes] = 8.17 ± 3.37 vs. Ks [*N. bombycis B* vs. *M. rotundata 7*] = 4.02 and Ks [*N. bombycis G* vs. *M. rotundata 7*] = 2.65) and *T. hominis* (mean Ks [*Har. saltator* genes vs. *T. hominis* genes] = 9.51 ± 1.81 vs. Ks [*T. hominis* vs. *Har. saltator 4*] = 0.19).

Manual inspections of contigs confirmed that all reported HTs were surrounded by genes arisen from vertical descent, and were located within contigs that were properly assembled (supplementary fig. S5, Supplementary Material online). All *An. algerae* TEs involved in phylogenetic inconsistencies were also found in other *An. algerae* strains that were cultured, sequenced, and assembled by others (see Materials and Methods).

## Discussion

### Origin of TE Diversity in the Microsporidia

The different LTR-retrotransposon families we detected in microsporidia genomes are all from a *Ty3/gypsy* group previously described from a wide variety of organisms, such as plants, fungi, and animals (Llorens et al. 2009). Three different subgroups appeared specific to the microsporidian species and independent from the well-described subgroups. The structure of these elements with one polyprotein instead of two ORFs for *gag* and *pol* makes them different from the rest of the *Ty3/gypsy* elements. They could represent the remnant of ancestral forms of LTR retrotransposons thought to have emerged from bacterial DNA transposons (Capy et al. 1996). These elements seem to have been particularly successful in *An. algerae* genome (40.90% of the total identified TE families), such a diversity is not observed in the other microsporidian genomes sequenced to date, even if *No. bombycis* and *Ed. aedis* showed quite large numbers of TEs families (table 1). DNA transposons represent the majority of TE sequences identified in the microsporidia (57.44% of the total identified TE families in this study), with three superfamilies being characterized (*Mariner/Tc1, Merlin,* and *piggyBac* superfamilies). Among the *Mariner/Tc1* elements, one microsporidian group does not appear to belong to any of the already described groups *Mariner*, *Tc1,* and *MaT*. As a cyanobacteria sequence is clustered within this group and as it has been hypothesized that these elements were derived from bacterial IS elements (Capy et al. 1996), this group could correspond to ancestral forms of *Mariner/Tc1* elements. The diversification of TE families indicates that the emergence of new families could be a recurrent process as there is evidence that TEs can be viewed as a mosaic of various elements that arose through recombination process (Capy et al. 1996; Lerat and Capy 1999; Auge-Gouillon et al. 2000). Alternatively, the hypothesis that these new TE families may originate from HTs not already characterized cannot be completely excluded. Hence, the large diversity of some TE families in the *An. algerae* genome could indicate that new TEs can easily be formed or gained in this species and possibly in other microsporidian genomes. This is particularly clear in the case of the *Merlin* DNA transposons whose numbers have drastically expanded in *An. algerae*. It has been proposed that the diversity and the size of TE families could be in part due to the population structure of the species where they are found like the population size (Jurka et al. 2011). In this case, the accumulation of TEs could be the result of genetic drift in small populations of these parasites, as microsporidian populations can be subject to genetic bottlenecks like any intracellular organism. Because genetic drift is associated with a decrease in the efficacy of natural selection, it is expected that deleterious mutations, as for example TE insertions, will be fixed in small populations. New TEs insertions could thus be fixed by genetic drift, which would lead to an increase of the genome size in the microsporidian species where they occur and offset genome reduction processes.

### Potential Impact of TEs on the Biology and Evolution of Microsporidia

Microsporidia are renowned for the genes they have lost, and genome sequencing of these parasites rarely demonstrates complexity that exceeds that found in free-living microbial eukaryotes. Here, however, we demonstrate that these organisms can harbor exaggerated numbers of TE families; matching those found in distant fungal symbionts and pathogens with genomes that are over twice as large (i.e., the plant symbiont *Laccaria bicolor* with171 TE families in a 60 Mb genome [Labbe et al. 2012]; the plant pathogen *Puccinia graminis* with 266 families in a 80 Mb genome) (Duplessis et al. 2011). The abundance of these elements in *An. algerae* genome (and to a lesser extent other members of this lineage) may reflect their pivotal role in the ecology and evolution of these parasites, as these elements are known contributors to genome plasticity in other organisms (Biemont 2010). This plasticity has also been linked with host–parasite interactions in distant relatives, most notably fungal and oomycete plant pathogens, so it is literally possible that TE abundance results in similar adaptive processes in microsporidia (Dean et al. 2005; Amyotte et al. 2012; Raffaele and Kamoun 2012). Recently, a SILAC (Stable Isotope Labeling by Amino Acids in Cell culture) approach allowed the detection of a Pol polyprotein among the parasite proteins during an infection process. A specific microsporidian regulation signal within the putative promoter of this TE suggests its domestication by the microsporidia, which may have provided an advantage in the evolutionary story of *An. algerae* to lure the host innate immune system (Panek et al. 2014). The fact that not all microsporidia share the same pool of TEs is in agreement with previous reports indicating that variation in the amount of TEs largely accounts for differences in genome size within this group—that is, bigger microsporidian genomes harbor more TEs (Williams et al. 2008; Corradi et al. 2009; Corradi and Slamovits 2011; Peyretaillade et al. 2012). The identification of many TEs families that are exclusive to microsporidia suggests their ancestral presence in these parasites, and many of these now continue to evolve through independent expansions and contractions in the different lineages.

### Evidence of Extremely Recent and Bidirectional HTs with Metazoans

Microsporidia benefited from HTs in many ways (reviewed in Selman and Corradi 2011), but to date only two of these have involved TEs (Heinz et al. 2012; Pan et al. 2013). Our study, however, shows that the role of TEs in the HT may have been overlooked in these parasites. Among the 17 phylogenetic incongruences identified, nine could conservatively be attributed to HTs, doubling the number of cases known for microsporidia. These elements appear to have been exchanged with various metazoan taxa, confirming previous reports based on one TE and two protein encoding genes (Selman et al. 2011; Heinz et al. 2012; Pombert et al. 2012). However, the number of cases we identified suggests that genetic exchanges with animals may be more frequent that previously appreciated. Interestingly, a few cases of HTs involved a planarian (*Sc. mediterranea*), a lineage that has never been reported to be infected by microsporidia. Indeed, prior to this study, *An. algerae* was only known to infect human and mosquito. Nevertheless, the very high sequence identity between several TEs from *An. algerae*, and, the very distantly related metazoans *Sc. mediterranea* and *D. ponderosae* suggest that the host range of some microsporidian species could be much larger than previously assumed. Host range is probably only partially known and identification of recent HTs could help us to refine it.

Most previous reports of HTs in microsporidia have involved sequences that are rather divergent (between 23% and 62%), suggesting that these events were rather ancient (Slamovits and Keeling 2004; Xiang et al. 2010; Selman et al. 2011; Cuomo et al. 2012; Heinz et al. 2012; Pombert et al. 2012; Nakjang et al. 2013; Pan et al. 2013), but in this study we found several compelling cases of HTs involving sequences sharing between 70% and 98% nucleotide identity. This elevated similarity suggests that microsporidia and metazoans taxa are actively exchanging TEs to this day, and particularly *piggyBac* elements. We also found cases where TEs appeared to have been donated by the microsporidia rather than being received. Our conservative analyses revealed two such cases, namely *Merlin7* and *Merlin10* (fig. 3), both of which involved the planaria *Sc. mediterranea.* Interestingly, this species has been documented to be involved in HTs in other occasions with Lepidoptera and vertebrates (Novick et al. 2010; Lavoie et al. 2013), so an intriguing possibility is is that latter HTs were mediated by a microsporidium and facilitated by the nonisolated germ-line of *Sc. mediterranea* (Schaack et al. 2010). If the phylogenetic inconsistencies we observed are real, and not the result of biases introduced by taxonomical sampling, the capacity of microsporidia to occasionally donate genetic material to other organisms would underscore their potential to act as “vectors” of genetic information in many environments, revealing completely new aspects of their biology with far-reaching consequences for our understanding of their biology.

## Supplementary Material

Supplementary file S1, figures S1–S5, and tables S1–S6 are available at *Genome Biology and Evolution* online (http://www.gbe.oxfordjournals.org/).

Supplementary Data
