# Supplementary material for: Microsporidian Genomes Harbor a Diverse Array of Transposable Elements that Demonstrate an Ancestry of Horizontal Exchange with Metazoans
Source: Genome Biol Evol. 2014 Aug 28;6(9):2289–300. doi: 10.1093/gbe/evu178 (PMC4202319; doi:10.1093/gbe/evu178)
Supplement: Supplementary Data [file supp_6_9_2289__index.html]

Microsporidian genomes harbour a diverse array of transposable elements that demonstrate an ancestry of horizontal exchange with metazoans — Microsporidian Genomes Harbor a Diverse Array of Transposable Elements that Demonstrate an Ancestry of Horizontal Exchange with Metazoans — Supplementary Data 

# Microsporidian Genomes Harbor a Diverse Array of Transposable Elements that Demonstrate an Ancestry of Horizontal Exchange with Metazoans

## Supplementary Data

files

**Files in this Data Supplement:**

- Supplementary Data - zip file
