# Supplementary figures and images for: Microsporidian Genomes Harbor a Diverse Array of Transposable Elements that Demonstrate an Ancestry of Horizontal Exchange with Metazoans
Source: Genome Biol Evol. 2014 Aug 28;6(9):2289–300. doi: 10.1093/gbe/evu178 (PMC4202319; doi:10.1093/gbe/evu178)

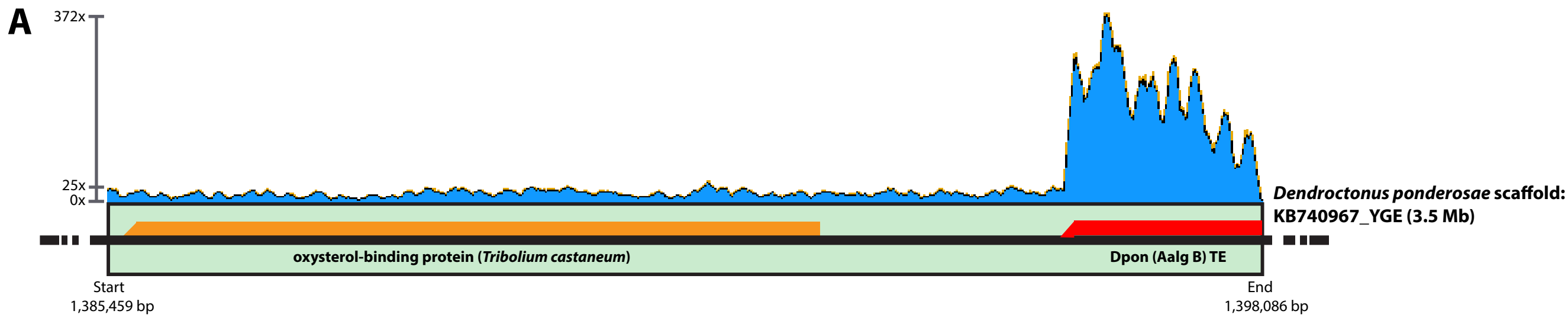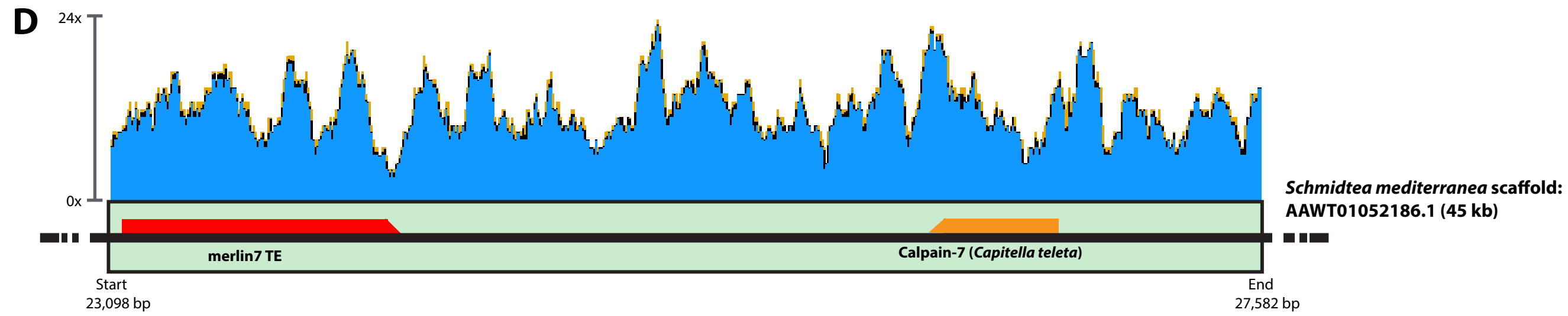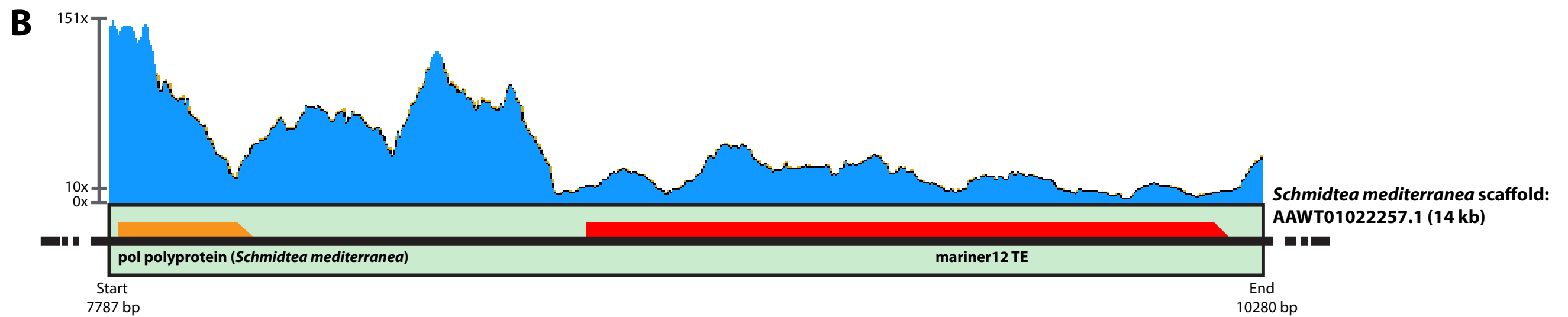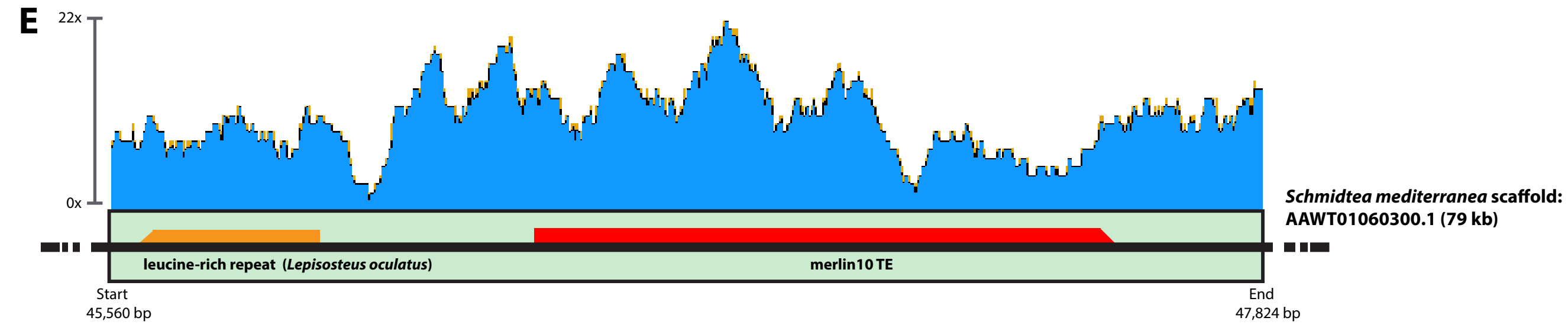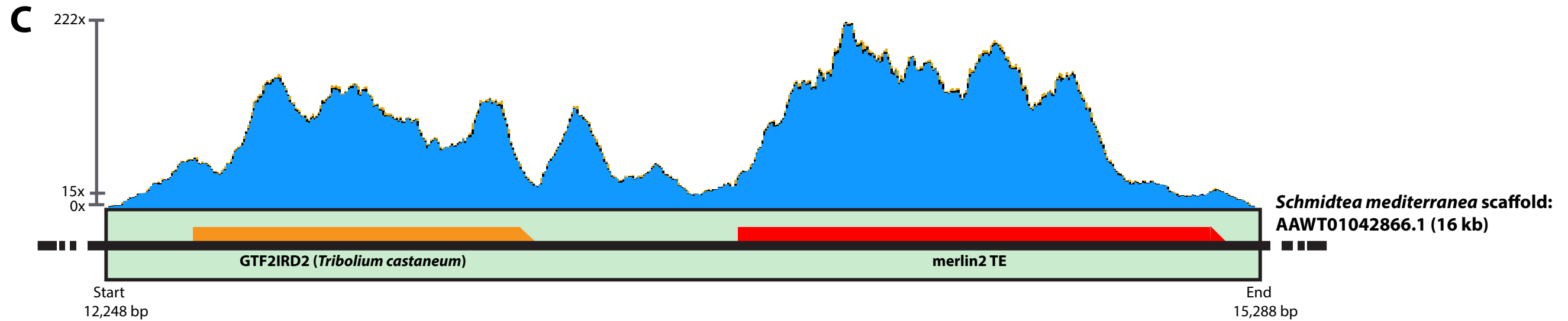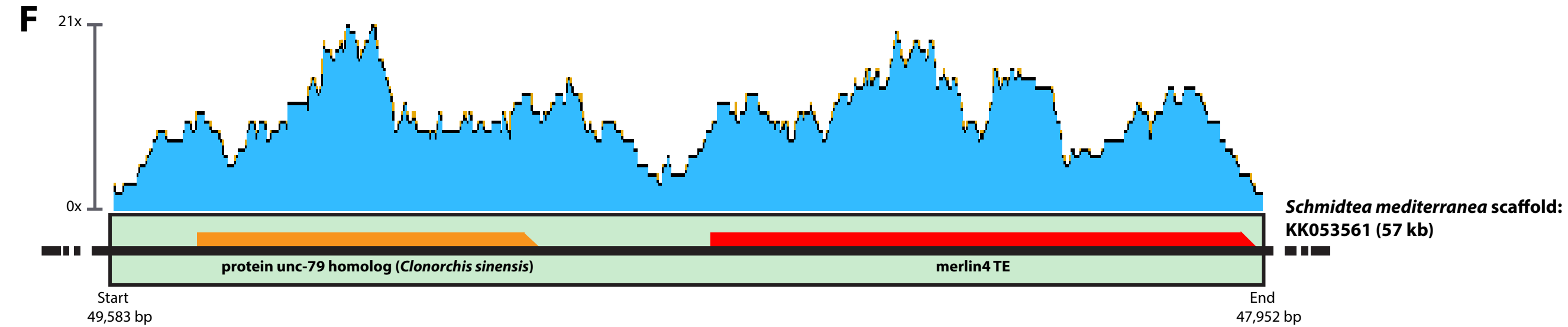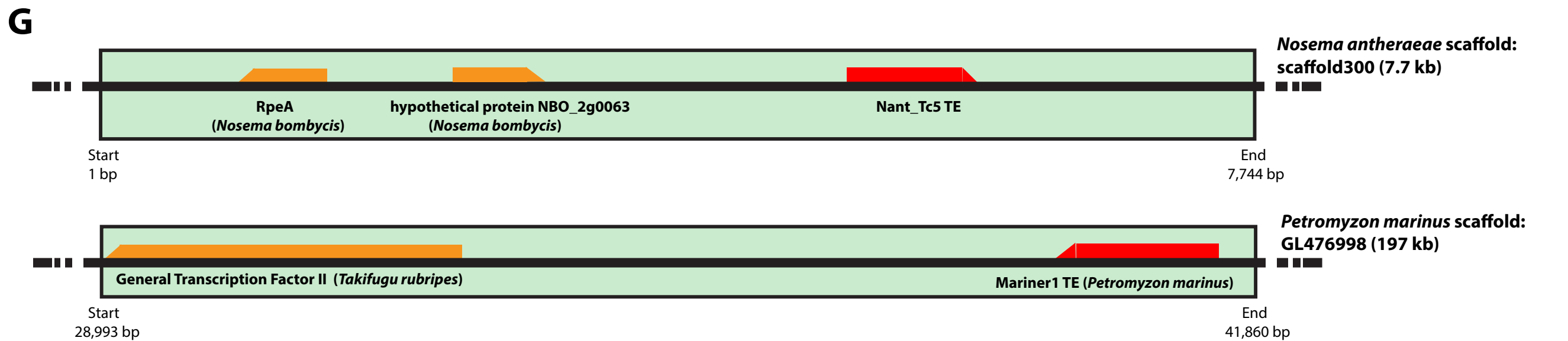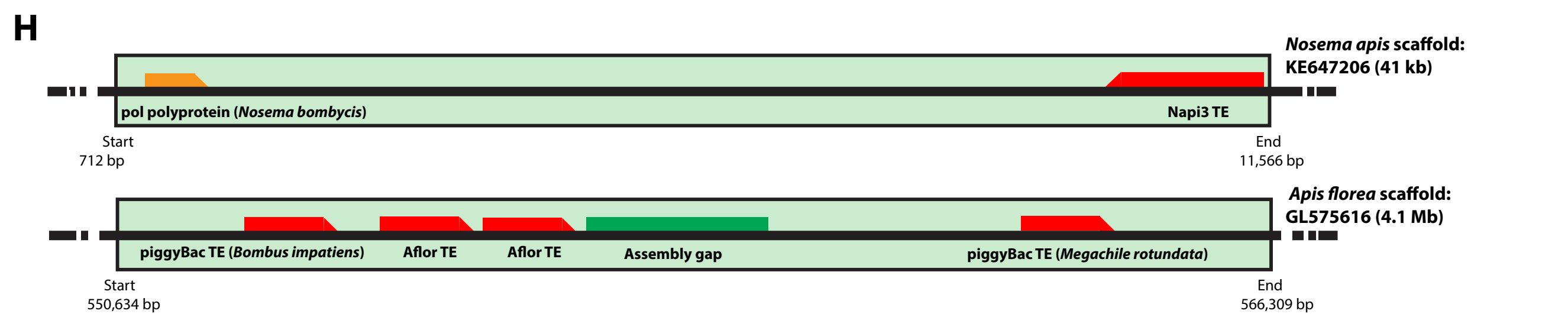

Supplement: Supplementary Data [file supp_evu178_suppl_data.zip › Supplementary Figure_S5.pdf]

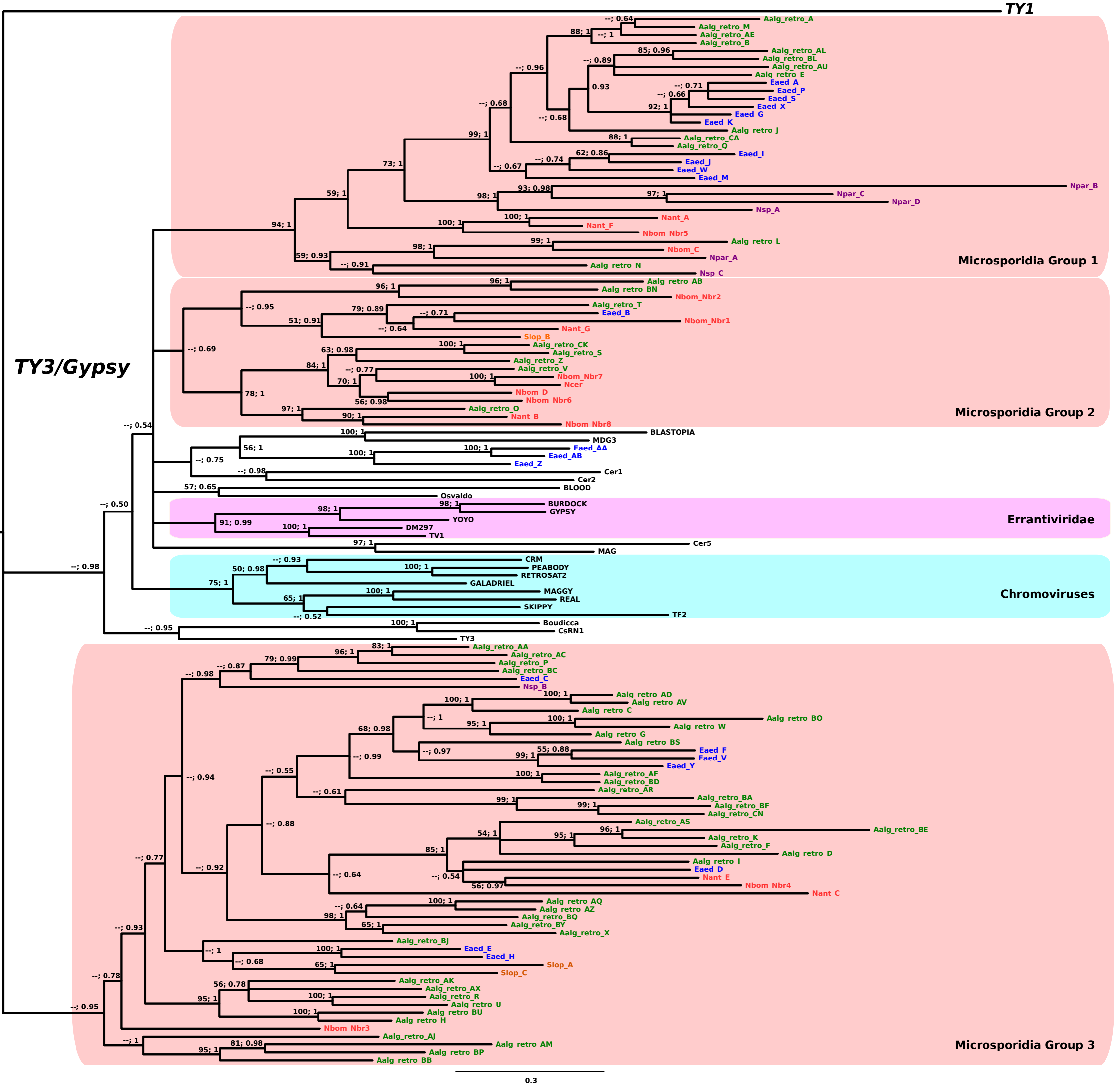

Supplement: Supplementary Data [file supp_evu178_suppl_data.zip › Supplementary_Figure_S1.pdf]

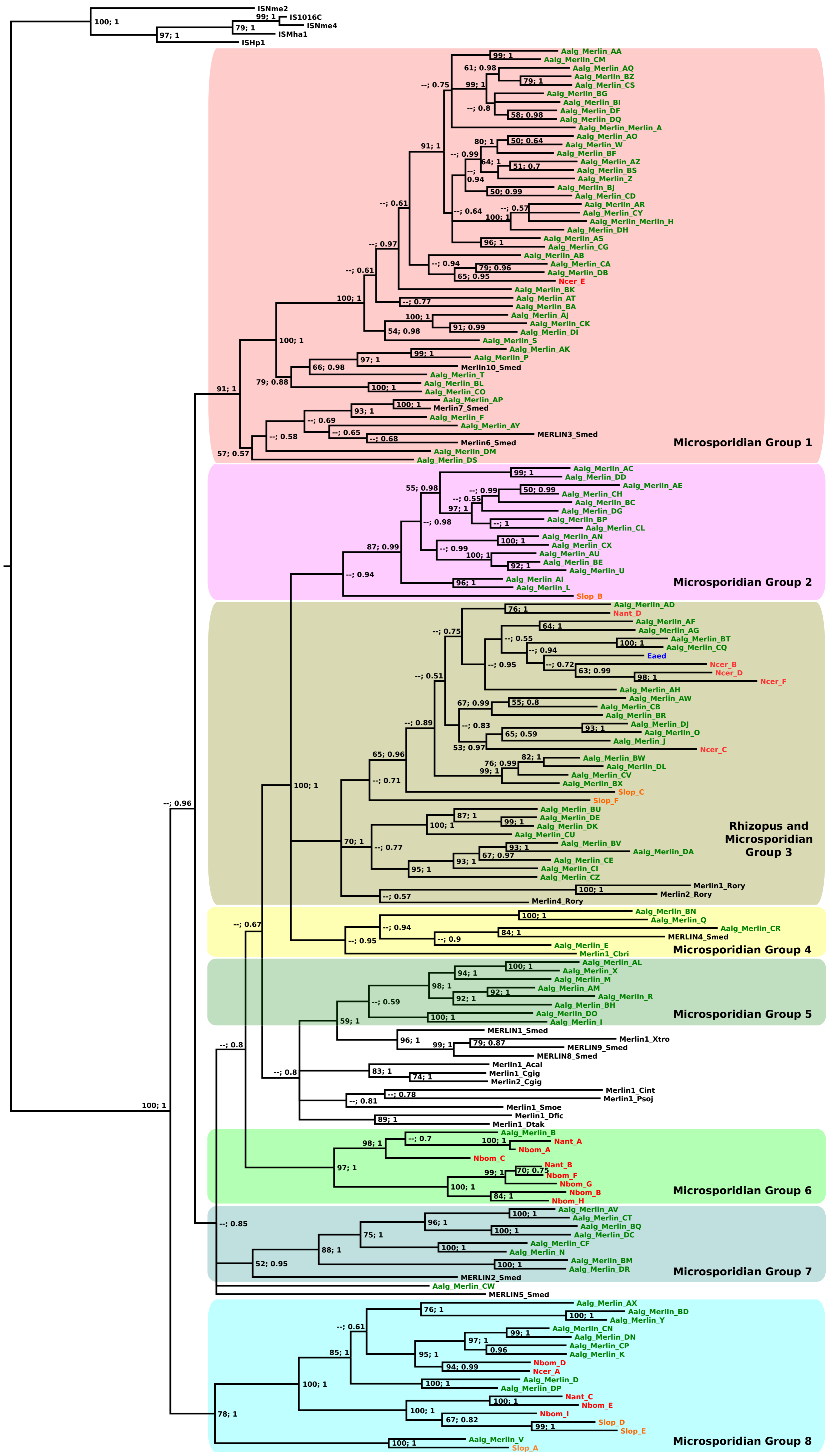

Supplement: Supplementary Data [file supp_evu178_suppl_data.zip › Supplementary_Figure_S2.pdf]

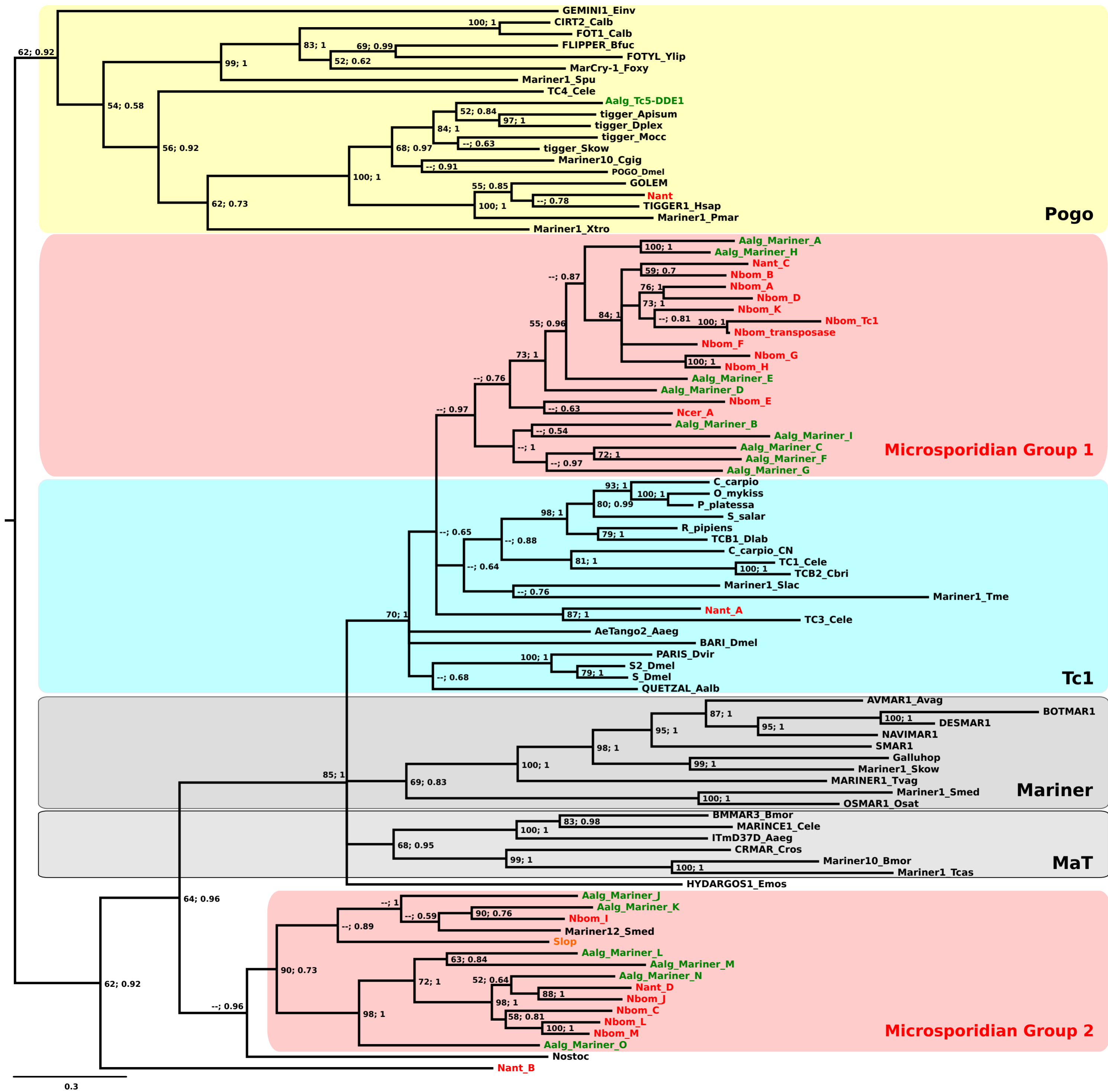

Supplement: Supplementary Data [file supp_evu178_suppl_data.zip › Supplementary_Figure_S3.pdf]

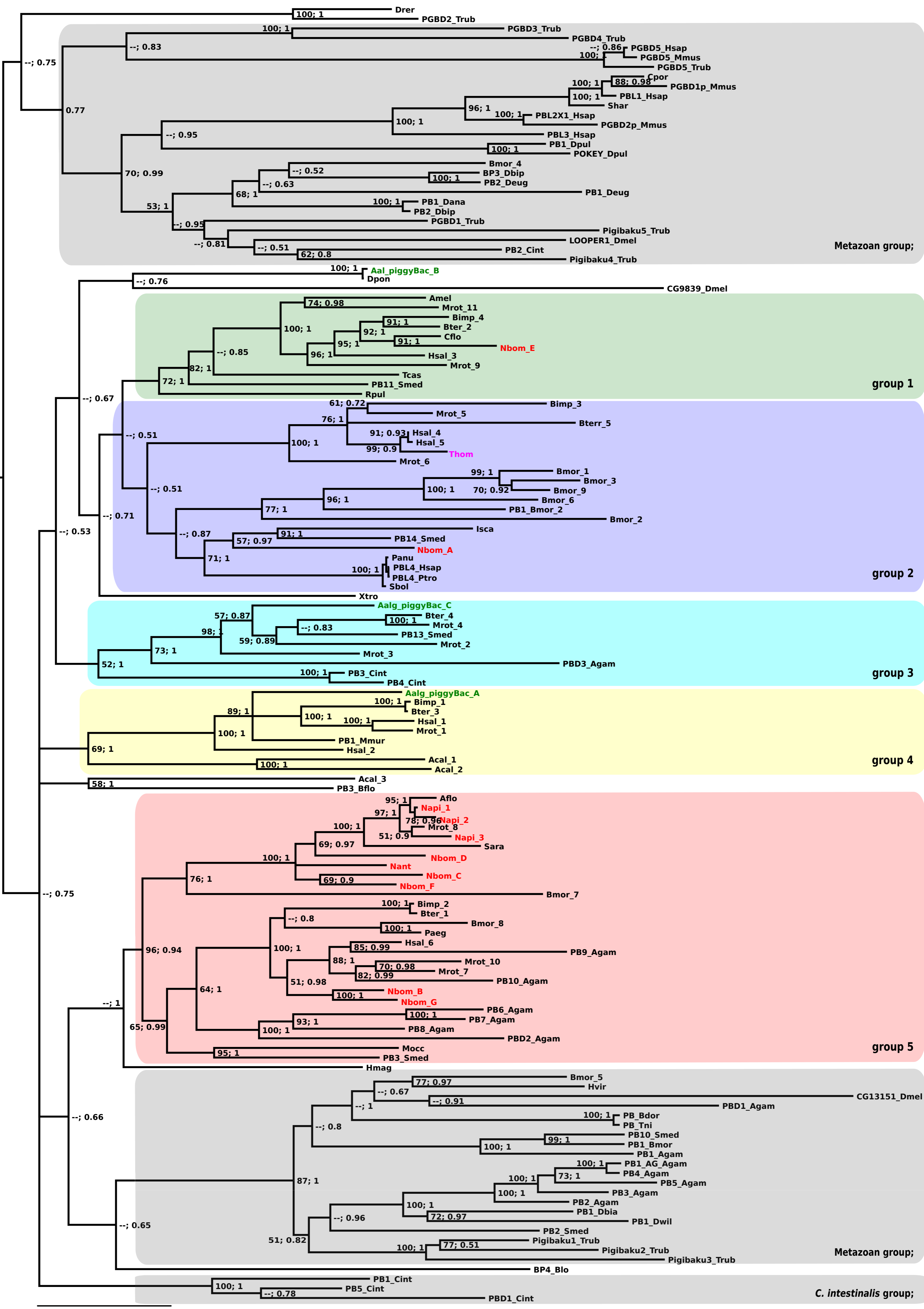

Supplement: Supplementary Data [file supp_evu178_suppl_data.zip › Supplementary_Figure_S4.pdf]
